# The Role of FOSL1 in Inflammatory Bowel Disease (IBD) Pathogenesis and IBD-Associated Tumorigenesis

**DOI:** 10.3390/biom16050647

**Published:** 2026-04-27

**Authors:** Grace J. Rilee, Senthil K. Radhakrishnan, Guang-Yu Yang, Jiong Li

**Affiliations:** 1 Department of Medicinal Chemistry, School of Pharmacy, Virginia Commonwealth University, Richmond, VA 23298-0540, USA; 2 Department of Pathology, Virginia Commonwealth University, Richmond, VA 23298-0540, USA; 3 Massey Comprehensive Cancer Center, Virginia Commonwealth University, Richmond, VA 23298-0540, USA; 4 Center for Drug Discovery, Virginia Commonwealth University, Richmond, VA 23298-0540, USA

**Keywords:** FOSL1, inflammatory bowel disease, colorectal cancer, pancreatic ductal adenocarcinoma, p53

## Abstract

Inflammatory bowel diseases (IBD), including ulcerative colitis and Crohn’s disease, are chronic inflammatory disorders of the gastrointestinal tract associated with epithelial barrier dysfunction, dysregulated immune responses, and an increased risk of cancer. Persistent inflammation is a key driver of IBD-associated tumorigenesis, yet the transcriptional regulators linking inflammatory signaling to epithelial transformation remain incompletely defined. FOSL1 (FOS-like antigen 1), a member of the activator protein-1 (AP-1) transcription factor family, has emerged as a critical mediator at the intersection of inflammation, epithelial homeostasis, and cancer progression. FOSL1 is induced by pro-inflammatory pathways commonly activated in IBD, including MAPK/ERK, NF-κB, and cytokine signaling, and regulates gene programs involved in cell proliferation, migration, barrier integrity, immune modulation, and survival. Accumulating evidence demonstrates that FOSL1 expression is elevated in inflamed intestinal mucosa and in IBD-associated malignancies, where it contributes to epithelial dysfunction, chronic inflammation, tumor initiation, metastasis, angiogenesis, and therapeutic resistance. Moreover, FOSL1-driven transcriptional networks show mechanistic overlap between IBD-associated colorectal cancer (CRC) and other inflammation-linked gastrointestinal cancers, such as pancreatic ductal adenocarcinoma (PDAC). In this review, we summarize current knowledge on the regulation and function of FOSL1 in intestinal inflammation and IBD-associated cancers, highlight its context-dependent roles in epithelial and immune compartments, and discuss emerging therapeutic strategies aimed at indirectly targeting FOSL1 signaling pathways.

## 1. Introduction

Inflammatory bowel diseases (IBD) are a group of chronic, relapsing inflammatory disorders of the gastrointestinal tract that consist primarily of ulcerative colitis (UC) and Crohn’s disease [1,2]. Although UC and Crohn’s disease share overlapping clinical and immunological features, these diseases differ in their distribution within the body and pathogenesis [3,4]. Crohn’s disease can affect any part of the gastrointestinal tract from the mouth to the anus and is characterized by transmural inflammation and skip lesions, whereas ulcerative colitis is limited to the colon and rectum and involves continuous mucosal inflammation [3]. The incidence and prevalence of IBD have increased globally over recent decades, and mortality remains low, with the most common cases reported between the ages of 15 and 35 years [5,6,7].

The pathogenesis of IBD is multifaceted and involves a complex interplay between genetic susceptibility, dysregulated immune responses, intestinal microbiota alterations, and the external environment [8]. Advances in genomic and microbiome research have identified numerous risk loci and microbial signatures associated with disease development and severity, while immunological studies have revealed aberrant activation of innate and adaptive immune pathways [9,10,11]. Therapeutic treatment has been focused on improving the disease and quality of life, reducing clinical side effects, and restricting the need for surgery [5]. The type of pharmaceutical treatment is prescribed based on the disease and stage, consisting of antibiotics, supportive medications, aminosalicylates, corticosteroids, and immunomodulators [5,12]. Despite the progress in therapeutic strategies, many patients do not respond or lose response to the available treatment, leaving many with immune suppression and an increased risk of developing cancer, mainly colorectal cancer (CRC) [12,13,14].

CRC arises from the accumulation of genetic and epigenetic alterations that promote abnormal epithelial proliferation and polyp formation within the colon and rectum. Although IBD accounts for only 1–2% of CRC cases, approximately 15% of IBD-related deaths are associated with CRC [15]. Chronic inflammation is a key driver of this increased risk and shares mechanistic parallels with inflammation-associated tumorigenesis in other gastrointestinal malignancies, including pancreatic ductal adenocarcinoma (PDAC), where sustained inflammatory signaling also promotes malignant transformation and tumor progression [16]. Treatment of colorectal cancer is multimodal and tailored to disease stage, tumor biology, and patient factors. Surgical resection remains the primary treatment for localized disease, often curative in early stages [17]. For metastatic disease, treatment typically involves a combination of chemotherapy, such as 5-fluorouracil (5-FU), radiation therapy, and targeted or immunotherapeutic agents, including anti-VEGF or anti-EGFR antibodies, selected according to molecular profiles (e.g., RAS/BRAF status) [17,18,19].

In contrast, PDAC represents one of the most aggressive solid malignancies, accounting for 90% of pancreatic cancer cases and a 5-year survival rate of 13% [20,21]. This poor prognosis is largely attributed to late-stage diagnosis, rapid disease progression, and resistance to conventional therapies, driven in part by a highly immunosuppressive microenvironment [20]. Interestingly, patients with IBD have a 45% increased risk of developing pancreatic cancer, potentially due to shared inflammatory pathways, immune dysregulation, and underlying genetic susceptibility [22,23]. Collectively, these observations underscore the broader impact of chronic inflammation in gastrointestinal cancers and highlight overlapping pathogenic mechanisms linking IBD to both CRC and PDAC.

FOSL1 (FOS-like antigen 1), a gene frequently overexpressed in colorectal cancer, is upregulated in response to inflammatory signaling pathways such as MAPK/ERK pathways, which are commonly activated in IBD [24]. This inflammation-driven activation of FOSL1 contributes to dysregulated epithelial proliferation, impaired barrier function, and altered wound-healing responses [25,26,27,28]. Notably, FOSL1 has also been implicated in pancreatic cancer, where it functions downstream of oncogenic KRAS to drive aggressive tumor behavior, epithelial-to-mesenchymal transition, and therapy resistance, highlighting shared inflammatory and transcriptional programs across gastrointestinal cancers [29].

In this review, we will focus on the context-dependent roles of FOSL1 in intestinal homeostasis, IBD, and IBD-associated malignancies. We will discuss the dual function of FOSL1 in epithelial repair and chronic inflammatory signaling, its emerging role in linking barrier dysfunction with immune activation, and its contribution to inflammation-associated tumorigenesis in CRC and PDAC. In addition, this review integrates recent mechanistic, transcriptomic, and therapeutic evidence to evaluate FOSL1 as both a biomarker and a potential target in IBD and related cancers. PubMed and Google Scholar were used as the primary search engines to identify relevant articles for this manuscript. Studies were included if they focused on one or more of the following areas: (1) FOSL1 in IBD, (2) FOSL1 in CRC, (3) FOSL1 in PDAC, and (4) the interaction of FOSL1 with p53 and other signaling pathways. Articles that mentioned FOSL1 but were not aligned with the central scope of this review were excluded. All literature included in this manuscript was published prior to 15 April 2026.

## 2. FOSL1

FOSL1 (FOS-like antigen 1), also known as FRA1 and a proto-oncogene, is a member of the activator protein-1 (AP-1) family of transcription factors, which regulate gene expression in response to a wide range of extracellular stimuli, including growth factors, cytokines, and cellular stress [27,30]. FOSL1 forms heterodimeric complexes primarily with JUN family proteins (c-JUN, JunB, and JunD), enabling binding to AP-1 consensus sequences within target gene promoters and enhancers [24,31,32]. Unlike other FOS family members, FOSL1 lacks a strong transactivation domain, and its activity is largely regulated at the post-translational level, particularly through phosphorylation-dependent stabilization [33,34]. FOSL1 expression can be rapidly induced in response to mitogenic and inflammatory signaling pathways [24].

FOSL1 plays a critical role in cellular processes such as proliferation, differentiation, migration, and survival, and has been extensively implicated in pathological contexts, especially cancer and inflammation [35,36]. Functionally, aberrant or sustained FOSL1 expression contributes to oncogenic programs by promoting epithelial–mesenchymal transition, extracellular matrix remodeling, and resistance to apoptosis across multiple tumor types [27,28,37,38,39]. In cancers such as CRC and PDAC, FOSL1 is frequently upregulated downstream of MAPK/ERK signaling, often through KRAS or BRAF mutations, where it enhances tumor progression, invasion, and inflammatory gene expression [35,40]. Additionally, FOSL1 has been linked to immune regulation, where it modulates cytokine production and cellular responses to inflammatory cues, further reinforcing its role at the intersection of inflammation and tumorigenesis [26,41,42].

Notably, FOSL1 also exhibits context-dependent tumor-suppressive functions. In certain settings, such as cervical cancer, FOSL1 overexpression has been shown to inhibit proliferation, induce apoptosis, and suppress metabolic reprogramming through activation of the STAT1-p53 signaling axis [43]. The contrasting role of FOSL1 highlights its complexity, suggesting that its function is highly dependent on cellular context, disease stage, and interacting molecular networks. These findings position FOSL1 as a multifaceted transcriptional regulator and a potential biomarker and therapeutic target in both malignant and inflammatory disorders, while emphasizing the need for context-specific strategies when considering its clinical targeting.

## 3. Role of FOSL1 in IBD and IBD-Associated Cancer

FOSL1 has emerged as an important regulator in inflammatory diseases such as IBD because of its central role in inflammation and immune signaling. In intestinal epithelial cells, FOSL1 regulates genes involved in barrier integrity, cell proliferation, and tissue remodeling, all of which are critical for maintaining mucosal homeostasis [24,36]. Dysregulated epithelial renewal and impaired barrier function are hallmarks of IBD, facilitating increased microbial translocation and immune activation.

Emerging evidence also suggests that FOSL1 may serve as an important link between microbiome-driven inflammation and host-microbe signaling. Microbial products such as lipopolysaccharide (LPS), flagellin, peptidoglycans, and short-chain fatty acids (SCFAs) can activate Toll-like receptors, MAPK/ERK, JNK, and NF-κB signaling pathways, many of which converge on AP-1 transcription factors, including FOSL1 [24,36,44,45]. Through these signaling networks, FOSL1 may regulate the expression of pro-inflammatory cytokines (TNF-α, IL-1β, and IL-6), chemokines, and epithelial barrier-associated genes, thereby influencing the balance between protective mucosal immunity and chronic inflammation [36,46]. Persistent dysbiosis may promote sustained FOSL1 activation, leading to exaggerated immune responses, defective epithelial repair, and progressive tissue damage [47,48]. Conversely, beneficial microbiota-derived metabolites such as butyrate and other postbiotics may indirectly suppress FOSL1-driven inflammatory signaling while restoring epithelial homeostasis, further supporting a role for FOSL1 as a mechanistic bridge between host-microbe interactions and chronic intestinal inflammation [49,50].

The expression of FOSL1-positive intestinal mucosa epithelial cells is significantly increased in patients with active IBD, possibly causing relapse and the progression to CRC [51,52]. In a study done by Wang et al. [51], FOSL1 was overexpressed in HCT-116 (116-FOSL1), a male colon cancer cell line derived from the colon, showing the role of FOSL1 in intestinal mucosal epithelial cell proliferation and damage repair. Overexpression of FOSL1 in HCT-116 cells resulted in a significant decrease in proliferative rate and damage repair in vitro, suggesting that FOSL1 interferes with intestinal mucosal epithelial barrier repair that is mediated through epithelial cell proliferation and migration. Furthermore, through Western blotting, it was shown that the overexpression of FOSL1 suppressed several pro-survival and anti-apoptotic proteins involved in a variety of inflammatory signaling pathways, such as Bcl-2, Survivin, Bcl-xL, and c-Myc. Notably, Survivin is a member of the inhibitor of apoptosis protein (IAP) family, while Bcl-2 and Bcl-xL belong to the Bcl-2 family of anti-apoptotic regulators, collectively contributing to cell survival and tissue repair [53]. The downregulation of these proteins suggests that FOSL1 may promote epithelial vulnerability by shifting the balance toward reduced survival signaling, thereby limiting effective mucosal regeneration following injury. Mechanistically, the overexpression of FOSL1 reduced STAT1, STAT3, STAT6, ERK1/2, and AKT expression. Taken together, these findings highlight FOSL1 as a potential negative regulator of intestinal epithelial repair and suggest its involvement in modulating inflammatory and apoptotic pathways that are critical for maintaining mucosal barrier integrity, thus causing relapse and potential for cancer progression.

Given the impact of FOSL1 on epithelial proliferation, survival, and inflammatory signaling pathways, its role in human intestinal inflammation has become an area of increasing interest. Accordingly, Sabzevary-Ghahfarokhi et al. [26] analyzed FOSL1 expression in ulcerative colitis, a major form of IBD, patients to assess its involvement in mucosal immune responses and epithelial barrier regulation, as well as examined its relationship with IL-11. In this cohort of 20 UC patients and 20 healthy controls, FOSL1 mRNA levels were significantly elevated in mildly inflamed colonic tissue compared to non-inflamed controls, suggesting an induction of FOSL1 under early inflammatory stress. This upregulation of FOSL1 in mild UC was accompanied by increased mucosal IL-11 protein levels, consistent with the known role of FOSL1/AP-1 in driving IL-11 expression in oxidative stress conditions [54,55]. The study also found a strong positive correlation between FOSL1 expression and IL-11 protein amounts in UC tissues, supporting the idea that FOSL1 may contribute to IL-11-mediated protective mechanisms in the inflamed gut. However, in severe UC, IL-11 protein levels were significantly reduced despite disease activity, and FOSL1 expression did not differ significantly from controls, potentially reflecting dysregulated epithelial stress responses or the effects of treatments such as corticosteroids on AP-1 signaling. These findings suggest the role of FOSL1 in UC pathophysiology, where its early upregulation may support mucosal defense and repair through IL-11 induction, but this response appears lost or ineffective in more severe inflammation, highlighting the complexity of FOSL1-driven pathways in IBD progression.

To investigate the role of FOSL1 with immune cells, Shetty et al. [56] used a comprehensive proteomic approach and mapped the interactomes of FOSL1, identifying a broad network of protein–protein interactions that are different from the normal AP-1 partners, such as JUN proteins, RNA-binding proteins, and transcriptional regulators, suggesting a complex program involved in Th17 function. Given that Th17 cells and their signature cytokines (e.g., IL-17A, 17F, IL-21, and IL-22) are mediators of inflammation in IBD [57], unraveling the interactions with FOSL1 could provide a framework for how it shapes pro-inflammatory T-cell responses that contribute to intestinal pathology. FOSL1 was shown to bind with other proteins to form regulatory complexes, including RUNX1, SIRT-1, and JUNB, to impair Th17 signaling. Notably, JUNB and RUNX1 interact with regulators (positive or negative) that control the fate of Th17, showing a context-dependent mechanism. FOSL1 was also observed to associate with activator and repressor complexes (e.g., HDAC2 and PCBP1) that control the lineage of Th17 cells, thus influencing the inflammatory cytokines produced.

Additionally, mounting evidence links FOSL1 to intestinal epithelial and immune responses; for example, FOSL1 is upregulated in colon tissues from ulcerative colitis patients and has been shown to modulate barrier integrity and inflammation in experimental colitis models through transcriptional regulation of matrix metalloproteinase-13 (MMP13), with knockdown of FOSL1 reducing inflammation and preserving tight junctions [27].

In the context of CRC, FOSL1 has been identified as a critical oncogenic driver with significant implications for tumor progression and metastasis. The study done by Liu et al. [28] demonstrated that FOSL1 expression is elevated in CRC tissues compared to adjacent normal tissues and correlates with poor patient prognosis, suggesting its potential as a prognostic biomarker. Silencing FOSL1 reduced proliferation, invasion, migration, epithelial-to-mesenchymal (EMT) abilities in vitro, and metastasis in vivo. Mechanistically, the authors elucidate a novel pathway in which FOSL1 upregulates the E3 ubiquitin ligase Smurf1, leading to ubiquitination and degradation of the tumor suppressor FBXL2. The loss of FBXL2 relieves inhibition of the Wnt/β-catenin signaling pathway, thereby promoting β-catenin nuclear translocation and activation of downstream oncogenic targets such as c-Myc and CCND1. Another study done by Liu et al. [58] showed that FOSL1 drives 5-fluorouracil (5-FU) resistance through transcriptional activation of PHLDA2, a downstream effector that promotes survival signaling, thereby decreasing cancer cell sensitivity to 5-FU. Functional assays confirmed that knocking down FOSL1 decreases PHLDA2 expression, suppresses tumor cell growth, and restores chemosensitivity, highlighting the FOSL1/PHLDA2 axis as a potential therapeutic target to overcome chemoresistance in CRC. FOSL1 has also been implicated in the angiogenic processes that support colorectal tumor growth. In CRC, FOSL1 and its downstream effector TIMP1 are both upregulated, and FOSL1 directly binds to the TIMP1 promoter to enhance its transcription. Elevated TIMP1, in turn, activates the VEGF signaling pathway, promoting proliferation and tube formation in endothelial cells, key steps in tumor-associated angiogenesis. Experimental silencing of TIMP1 not only suppresses endothelial cell proliferation and angiogenesis in vitro but also attenuates the angiogenic effects driven by FOSL1 overexpression, indicating that the FOSL1/TIMP1/VEGF axis is a critical mediator of vascular growth in colon carcinoma [59].

Similarly to CRC, FOSL1 expression was found to be elevated in PDAC tissues and correlated with poor patient survival, highlighting its clinical relevance [60]. In a study done by Dai et al. [61], SMAD4 directly suppresses FOSL1 expression, and a loss of SMAD4 activates FOSL1 expression and enhances metastatic potential. Functional experiments demonstrated that FOSL1 is necessary to drive metastatic colonization to the lung, independent of effects on primary tumor growth. Additionally, recent work has uncovered a cooperative FOSL1 transcriptional mechanism in PDAC metastasis [62]. In PDAC cells, combined stimulation with TNFα and epidermal growth factor (EGF) enhances co-occupancy of FOSL1 and p65 at specific enhancer regions, leading to the activation of genes associated with cell migration and motility. Chromatin immunoprecipitation and motif analyses revealed that FOSL1 and p65 co-bind regulatory elements enriched for both AP-1 and NF-κB motifs, and that this cooperative binding is linked to epigenetic activation of a pro-metastatic transcriptional program. Physical interactions between FOSL1 and p65 are strengthened by combined TNFα and EGF signaling, and this partnership facilitates RNA polymerase II recruitment to target genes, promoting their expression. Most recently, FOSL1 has been shown to promote the malignant progression of PDAC by interacting with HMGA1 to transcriptionally regulate the expression of genes related to cancer stemness, EMT, and multidrug efflux. When HMGA1 underwent knockdown, stemness-, EMT-, and multidrug efflux-related genes were inhibited, thus preventing the metastasis and progression of PDAC [60].

The NF-κB signaling pathway is persistently activated and plays a central role in driving pro-inflammatory gene expression in immune and epithelial cells, promoting pro-tumorigenic and pro-inflammatory states in IBD and IBD-associated cancers [63]. p53 is a protein known to be a tumor suppressor, and p53 abnormalities can be seen in more than 50% of human tumors [64]. In a comprehensive review by Talotta et al. [65], they highlight not only the regulatory circuits controlling FOSL1 expression and protein stability, but also a model where p53 and FOSL1 engage in regulatory interactions. In cells with functional, wild-type p53, FOSL1 expression can be reduced through p53-dependent induction of specific miRNAs (such as miR-34 family members) that directly target FOSL1 mRNA, thereby suppressing FOSL1-driven pro-invasive programs and EMT gene networks. Conversely, loss of p53 function or the presence of mutant p53 activates FOSL1, leading to enhanced FOSL1 expression and AP-1 transcriptional activity that drives metastasis, survival, inflammation, and therapy resistance. These results were seen in HCT116 cells treated with nutlin-3a, an antagonist of the MDM2-p53 interaction. Additionally, FOSL1 itself is involved in stress and survival pathways that intersect with p53-regulated pathways. In another study by Vallejo et al. [40], the genetic mouse models used (KrasLSL-G12D; Trp53f/f) incorporate loss of wild-type p53, indicating that FOSL1 upregulation and its tumor-promoting effects occur in a p53-deficient background. In this setting, the absence of functional p53 likely removes key constraints on proliferative and survival pathways, permitting FOSL1 to drive transcriptional programs. Furthermore, deletion of FOSL1 in the p53-deficient background significantly diminishes tumor burden and extends survival in vivo. Broader literature supports the concept that p53 loss or mutation can activate FOSL1 expression and activity in various cancers, and that mutant p53 proteins can even cooperate with other transcription factors to drive FOSL1 transcription in contexts such as metastatic progression [37,43,66]. In context, disruption of upstream regulators linked to p53 signaling can alleviate p53-dependent suppression of FOSL1, resulting in increased expression of FOSL1 and malignant progression, such as in breast cancer models where JNK2 has been shown to support p53 stability and transcriptional activity. When the JNK3-p53 axis is defective, p53-dependent suppression of FOSL1 is alleviated, resulting in elevated FOSL1 levels, which promote tumor growth and metastasis [67]. Additionally, in breast carcinoma, mutant p53 has been demonstrated to cooperate with the ETS family transcription factor ELK1 at the FOSL1 promoter, enhancing FOSL1 transcription. This cooperative interaction increases AP-1 activity and drives EMT-associated gene expression, invasion, and metastatic progression [66]. In contrast, in a study done by Zhang et al. [43], FOSL1 functions as a tumor suppressor in cervical cancer cells. Mechanistically, FOSL1 upregulates STAT1, which enhances p53 signaling, leading to suppressed cell growth and increased apoptotic activity.

## 4. Treatments and Therapies of Targeting FOSL1 in IBD and IBD-Associated Cancers

Collectively, these studies establish FOSL1 as a regulator of IBD and IBD-associated cancers, highlighting FOSL1 as a promising therapeutic target. Despite strong evidence implicating FOSL1 as a critical transcriptional regulator of tumor progression, invasion, and therapy resistance, direct pharmacologic targeting of FOSL1 remains at an early stage, and no FOSL1-selective agents are currently approved for the treatment of UC, CD, CRC, or PDAC. One major limitation is that FOSL1 is a transcription factor, a class of proteins historically considered difficult to drug due to the lack of enzymatic activity and well-defined ligand-binding pockets [24,35]. Nevertheless, elevated FOSL1 expressions have been consistently associated with poor prognosis, aggressive tumor phenotypes, and metastatic behavior in CRC and PDAC, underscoring its attractiveness as a therapeutic target and motivating the development of alternative, indirect targeting strategies.

Recent advances in targeting FOSL1 have largely emerged from preclinical and experimental studies, particularly through nucleic acid-based approaches. A study done by Maietta et al. [68] shows a promising dual-therapy strategy for PDAC that targets stromal and transcriptional drivers by combining sequential siRNA-mediated silencing of YAP1 and FOSL1 with epigenetic modulation and conventional chemotherapy. Using liposomal siRNA complexes to knockdown YAP1 and FOSL1, two transcription factors implicated in metastasis, inflammation, and resistance [36,69,70], cancer-associated fibroblast activity and stromal density were reduced, thereby improving penetration and efficacy of entinostat (a histone deacetylase inhibitor) with gemcitabine. The data suggest that combining targeted gene silencing with epigenetic therapy can remodel the tumor microenvironment and sensitize PDAC to treatment. In parallel, indirect regulation of FOSL1 through tumor-suppressive microRNAs, such as miR-34a, has been shown to reduce FOSL1 expression and invasive behavior in colorectal cancer models [35,71], further supporting the ability of suppressing FOSL1-driven programs without directly inhibiting the protein itself.

Another potential strategy involves the repurposing of small-molecule drugs to indirectly target FOSL1 activity. Although there are currently no highly selective small-molecule inhibitors that effectively and specifically block FOSL1 transcriptional function in vivo, several experimental and repurposed compounds have been shown to modulate FOSL1 expression or reduce AP-1-dependent transcription, particularly in inflammatory and immune-related contexts. Fanjul et al. [72] screened 50 synthetic retinoids and found SR11302 selectively inhibits AP-1 transcription without activating retinoic acid receptors (RARs). In an ex vivo 4D lung cancer model, SR11302 was able to reduce metastatic lesion formation [73]. SR11302 has also been used in vivo to dampen T-cell activation and inflammatory responses, in part through suppression of AP-1 complexes that include FOSL1 [36], illustrating that pharmacologic modulation of pathways upstream or cooperative with FOSL1 may influence its activity and downstream oncogenic programs. Additionally, Zaman et al. [74] showed that the small molecule AP-1 inhibitor T-5224, originally developed to inhibit FOS/JUN transcriptional activity, binds directly to the FOSL1/JUN complex and disrupts its DNA binding in head and neck squamous cell carcinoma (HNSCC) cancer stem cells (CSCs). Using T-5224 as the warhead, they were able to design and synthesize a series of PROTACs that recruit the cereblon (CRBN) E3 ligase to induce proteasomal degradation of FOSL1. Among these, compound 4 markedly suppressed tumor growth, eliminated lineage-traced CSCs, and improved survival in vivo, achieving an estimated 30–100 fold increase in efficacy over T-5224. Niidome et al. [75] discovered that the antiepileptic drug levetiracetam (LEV) can suppress FOSL1 expression and AP-1 transcriptional activity in microglia, resulting in decreased pro-inflammatory cytokine expression and attenuated neuroinflammation. While these findings were obtained outside the gastrointestinal system, they provide proof-of-concept that clinically approved drugs can influence FOSL1-dependent transcriptional programs and may be candidates for repurposing in chronic inflammatory diseases, including IBD.

Beyond direct inhibition, therapeutic strategies may focus on upstream regulators or interacting pathways that influence FOSL1 expression and function. In cancer biology, where FOSL1 is a well-established oncogenic driver, researchers are exploring combination strategies that disrupt FOSL1-dependent transcriptional networks. This includes looking at the MAPK/ERK signaling, KRAS-driven oncogenic programs, and inflammatory pathways mediated by NF-κB p65. There are several classes of targeted anticancer drugs that act upstream of FOSL1 and could possibly modulate FOSL1 expression or activity indirectly by suppressing signaling pathways that drive AP-1 family transcription factors. MAPK/ERK pathway inhibitors such as the MEK1/2 inhibitors trametinib, cobimetinib, and binimetinib are approved or under clinical evaluation for cancers with aberrant RAS/RAF signaling; these agents block MEK-mediated activation of downstream effectors that help drive AP-1 (including FOSL1) transcriptional programs, and they have shown antitumor activity in multiple solid tumors, including RAS/MAPK-driven malignancies like CRC and PDAC model systems [76]. KRAS inhibitors, like sotorasib and adagrasib, block a key driver of MAPK/ERK signaling, reducing downstream transcriptional activation that would otherwise elevate AP-1 and FOSL1-associated gene expression [35,77]. In a study done by Zhou et al. [78], they found BI 1701963 (SOS1 inhibitor) prevented KRAS activation, further dampening oncogenic RAS/MAPK signaling that correlates with FOSL1 induction in KRAS-driven tumors. While these agents do not directly bind FOSL1, preclinical evidence indicates that disruption of upstream MAPK or KRAS signaling can blunt the transcriptional networks that sustain FOSL1 expression and its cooperation with other transcription factors [79]. In addition to MAPK/ERK and KRAS targeting, NF-κB pathway modulation has been explored in cancer therapy because NF-κB p65 cooperates with AP-1 factors like FOSL1 in controlling pro-inflammatory and pro-tumorigenic gene networks, particularly in inflammation-associated cancers [62]. Even though highly selective small-molecule NF-κB p65 inhibitors are not yet widely approved, pharmacologic inhibition of NF-κB signaling using IKK (IκB kinase) inhibitors or anti-inflammatory agents can reduce activation of NF-κB/AP-1 transcriptional programs and has been shown in preclinical models of pancreatic and colorectal cancers to induce tumor cell apoptosis and sensitize tumors to other therapies [80]. Such strategies emphasize the potential of combination therapies that inhibit MAPK/RAS and NF-κB signaling to indirectly suppress FOSL1-centered transcription in IBD, CRC, PDAC, and potentially other FOSL1-driven cancers.

In the context of IBD (UC and CD), emerging evidence supports postbiotics as a promising therapeutic strategy. Unlike probiotics, postbiotics offer improved safety, stability, and reduced risk of microbial translocation, making them particularly attractive in conditions such as IBD where barrier integrity and the immune system are compromised [81,82]. Postbiotics—including short-chain fatty acids (SCFAs), vitamins, bacteriocins, microbial metabolites, and cell wall components—have emerged as promising adjuncts in IBD therapy by exerting immunomodulatory and barrier-protective effects, thereby serving as critical mediators between the gut microbiota and the host immune system to restore intestinal homeostasis [82,83]. Given that many microbiota-derived signals converge on inflammatory pathways such as MAPK, NF-κB, and STAT3, which are also known upstream regulators of FOSL1, it is possible that some of the beneficial effects of postbiotics are mediated through indirect suppression of FOSL1-driven inflammatory signaling [24].

Preclinical and translational studies further demonstrate that postbiotics can attenuate intestinal inflammation, repair mucosal damage, and stabilize the gut microbial environment, with effects comparable to or, in some cases, more consistent than probiotics due to their defined composition. In a preclinical study done by Chen et al. [49], the administration of sodium butyrate improved survival rate, body weight, colon length, and decreased clinical score on 2,4,6-trinitrobenzene sulfonic acid-induced colitis mice through inhibiting the inflammatory response of macrophages. Similar results could be seen with DSS-induced colitis mice with sodium butyrate and butyrate derivatives [84]. Beyond SCFAs, bacterial lysates and inactivated probiotics also demonstrate therapeutic potential. Cell wall components from *Lactobacillus casei*, *Lactobacillus acidophilus*, and *Lactobacillus rhamnosus* have been shown to protect against LPS-induced colitis by suppressing pro-inflammatory cytokines (TNF-α, IL-6, IL-1β) while increasing anti-inflammatory IL-10 levels, reflecting strong immunomodulatory effects. These treatments additionally reduce oxidative stress and improve antioxidant defenses, leading to enhanced tissue integrity and reduced inflammation, with *Lactobacillus acidophilus* showing the most pronounced benefit [85]. Translationally, improved clinical outcomes of UC patients were seen with rectal administration of butyrate while suppressing NF-κB activation in colonic lamina propria macrophages, reflecting reduced mucosal inflammation. In a separate study, Segain et al. [86] demonstrated that oral administration of sodium butyrate in CD patients suppressed NF-κB signaling, leading to a reduction in pro-inflammatory cytokine expression. Notably, because NF-κB signaling promotes FOSL1 expression and stability, while FOSL1 can further amplify NF-κB activity, these findings suggest that butyrate-mediated inhibition of NF-κB may indirectly disrupt this pro-inflammatory feedback loop and reduce pathogenic FOSL1 activation during chronic intestinal inflammation.

More studies further support the therapeutic potential of dietary bioactive compounds in ulcerative colitis through their effects on inflammation, epithelial barrier integrity, and host-microbe interactions. The human milk oligosaccharide 2′-fucosyllactose (2′-FL) was shown to significantly alleviate DSS-induced colitis, restore tight junction structure, and enhance expression of barrier-associated proteins such as ZO-1 and occludin. Mechanistically, these protective effects were mediated through inhibition of STAT3 palmitoylation and phosphorylation, leading to reduced inflammatory cytokine production and improved mucosal repair [87]. Similarly, the flavonoid neohesperidin attenuated DSS-induced ulcerative colitis by suppressing MAPK/NF-κB signaling, lowering pro-inflammatory cytokine levels, and restoring barrier proteins, including Claudin-3 and ZO-1. In addition, neohesperidin favorably altered gut microbiota composition by increasing beneficial bacterial genera such as *Allobaculum*, *Bacteroides*, and *Sutterella* [88]. Because STAT3, MAPK, and NF-κB pathways all contribute to the transcriptional activation and persistence of FOSL1, these findings raise the possibility that compounds such as 2′-FL and neohesperidin may also exert some of their protective effects through indirect modulation of FOSL1 activity. This potential connection further supports the concept that FOSL1 may function as a key downstream effector linking microbial metabolites, epithelial barrier dysfunction, and chronic inflammation in IBD.

Together, these approaches reflect a growing area of research positioning FOSL1 as a clinically relevant node at the intersection of inflammation, epithelial barrier dysfunction, microbiome-driven responses, and cancer progression. Although direct anti-FOSL1 therapies for IBD have not yet been developed, accumulating mechanistic evidence suggests that indirect modulation of FOSL1 through pathways such as MAPK, NF-κB, STAT3, JNK, and host–microbe signaling may offer therapeutic benefit for IBD and IBD-associated cancers. These emerging strategies provide an important foundation for future translational efforts aimed at selectively targeting pathogenic FOSL1 activity while preserving its protective roles in epithelial repair and mucosal homeostasis.

## 5. Discussion and Challenges

**Figure 1:**
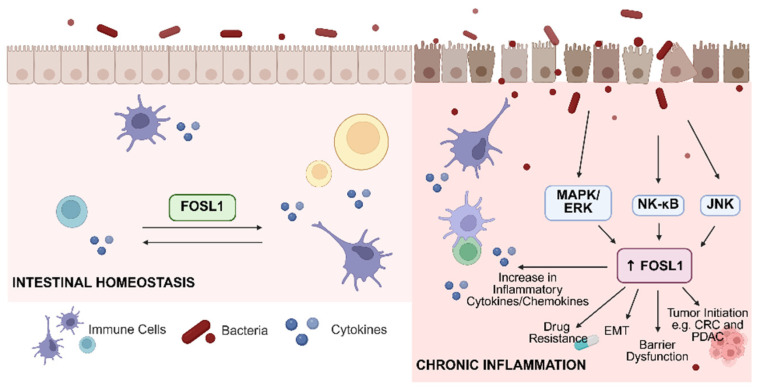
Role of FOSL1 in intestinal homeostasis and chronic inflammation. During homeostasis, FOSL1 regulates barrier maintenance and controls inflammation. Dysregulated or sustained expression of FOSL1 through inflammatory pathways can increase the production of inflammatory cytokines, cause drug resistance, EMT, damage tight junctions, and create an environment where tumors can form. (created using BioRender).

FOSL1, a component of the AP-1 transcription factor complex, has emerged as a key regulator of intestinal homeostasis and inflammatory signaling in IBD (Figure 1). Under physiological or acute stress conditions, FOSL1 contributes to epithelial integrity by regulating genes involved in barrier maintenance, wound repair, and controlled immune responses, thereby supporting mucosal adaptation to transient injury [89,90,91]. In intestinal epithelial cells, FOSL1 influences the expression of tight junction-associated proteins, including occludins, claudins, and zonula occludens proteins, which are essential for maintaining selective barrier permeability [92]. For example, in breast cancer, miRNA-130a can reduce FOSL1 levels, where FOSL1 promotes the expression of tight junction protein ZO-1. Because of this, miRNA-130a indirectly inhibits ZO-1 through FOSL1, inhibiting metastasis and migration [93]. Beyond tight junction regulation, FOSL1 is also involved in epithelial restitution by promoting cell migration, proliferation, and wound closure following mucosal injury [89,94]. Studies have shown that FOSL1 is upregulated after nerve injury, where it promotes Schwann cell proliferation and migration, thereby supporting nerve regeneration and repair [94]. Furthermore, Mao et al. [89] demonstrated that FOSL1 is a key regulator of skin wound healing, promoting keratinocyte-driven repair by activating the IL-17 signaling pathway. These functions are particularly important during acute inflammatory stress, where rapid epithelial repair is necessary to restore barrier integrity.

Dysregulated FOSL1 activity may therefore contribute to increased epithelial leakiness and microbial translocation, both of which are characteristic features of IDB. This is often driven by persistent MAPK/ERK signaling, shifting its role toward a pathogenic state, promoting the transcription of pro-inflammatory cytokines and chemokines that amplify immune cell recruitment and chronic inflammation [24,95,96]. A study done by Liang et al. [97], FOSL1 was able to promote psoriatic keratinocyte hyperproliferation and inflammation by transcriptionally activating TRAF3 and thereby enhancing NF-κB–mediated NLRP3 inflammasome signaling. The knockdown of FOSL1 reduced NLRP3 inflammasome activation and inflammatory cytokines (IL-6, IL-8, CCL17). In IBD, this transition is particularly significant, as elevated FOSL1 expression observed in both preclinical models and patient-derived transcriptomic datasets is associated with intestinal inflammation, epithelial barrier dysfunction, disease progression, and increased risk of tumor initiation, including CRC and PDAC. Studies have shown that FOSL1 expression is significantly increased in inflamed colonic mucosa of UC patients and is positively associated with IL-11 levels [26]. Collectively, these findings underscore the multifaceted and context-dependent nature of FOSL1, positioning it as a molecular switch that balances protective epithelial responses with chronic inflammatory signaling, and highlighting its central role in IBD pathogenesis.

Despite growing mechanistic insight, translating FOSL1 biology into therapeutic strategies for IBD and IBD-associated cancers remains challenging. FOSL1 has been historically difficult to target directly with small molecules. Current evidence instead supports indirect approaches, such as modulating upstream signaling pathways, downstream effector genes, or interacting transcriptional networks that converge on FOSL1 activity. Preclinical studies demonstrating reduced colitis severity following FOSL1 knockdown provide proof-of-concept that targeting this pathway may be beneficial [27]; however, the dual roles of FOSL1 in non-disease versus pathogenic responses raise important concerns regarding timing, disease stage, and cell specificity. Therapeutic inhibition of FOSL1 may be advantageous in settings of chronic inflammation driven by immune activation, while preserving or restoring its epithelial stress-response functions may be critical for mucosal healing and carcinogenesis.

## 6. Conclusions

FOSL1 has emerged as an important regulator of intestinal homeostasis, inflammation, epithelial barrier integrity, and tumorigenesis. Although transient FOSL1 activation may support epithelial repair, wound healing, and mucosal adaptation to injury, sustained or dysregulated signaling can promote chronic inflammation, barrier dysfunction, immune activation, and progression toward malignancies like CRC and PDAC. Importantly, the diverse functions of FOSL1 show its highly context-dependent nature. In some settings, FOSL1 drives inflammatory signaling and tumor progression, while in others, it has more suppressive effects. The complexity presents opportunities for novel therapeutic development, whether that be through direct inhibition of FOSL1 or indirect strategies like targeting upstream signaling pathways, microbiome-derived mediators, or downstream effector genes. A deeper understanding of how FOSL1 integrates with inflammatory, microbial, and epithelial signals will help provide more precise interventions for IBD and IBD-associated cancers.

## Data Availability

The original contributions presented in this study are included in the article.
